# Nanomotion Spectroscopy as a New Approach to Characterize Bacterial Virulence

**DOI:** 10.3390/microorganisms9081545

**Published:** 2021-07-21

**Authors:** Maria I. Villalba, Leonardo Venturelli, Ronnie Willaert, Maria E. Vela, Osvaldo Yantorno, Giovanni Dietler, Giovanni Longo, Sandor Kasas

**Affiliations:** 1 Laboratory of Biological Electron Microscopy, Ecole Polytechnique Fédérale de Lausanne (EPFL), 1015 Lausanne, Switzerland; ines.villalba@epfl.ch (M.I.V.); venturelli.leonardo@gmail.com (L.V.); giovanni.dietler@epfl.ch (G.D.); 2 Centro de Investigación y Desarrollo en Fermentaciones Industriales (CINDEFI), Facultad de Ciencias Exactas, Universidad Nacional de La Plata-CONICET, 1900 La Plata, Argentina; yantorno@quimica.unlp.edu.ar; 3 Research Group Structural Biology Brussels, Alliance Research Group VUB-UGent NanoMicrobiology (NAMI), 1050 Brussels, Belgium; Ronnie.Willaert@vub.be; 4 International Joint Research Group VUB-EPFL BioNanotechnology & NanoMedicine, 1050 Brussels, Belgium; 5 Instituto de Investigaciones Fisicoquímicas Teóricas y Aplicadas (INIFTA), Universidad Nacional de La Plata-CONICET, 1900 La Plata, Argentina; mevela@gmail.com; 6 Istituto Di Struttura Della Materia–CNR, 00133 Roma, Italy; giovanni.longo@artov.ism.cnr.it; 7 Centre Universitaire Romand de Médecine Légale, UFAM, Université de Lausanne, 1015 Lausanne, Switzerland

**Keywords:** nanomotion, bacteria, AFM, *B. pertussis*

## Abstract

Atomic force microscopy (AFM)-based nanomotion detection is a label-free technique that has been used to monitor the response of microorganisms to antibiotics in a time frame of minutes. The method consists of attaching living organisms onto an AFM cantilever and in monitoring its nanometric scale oscillations as a function of different physical-chemical stimuli. Up to now, we only used the cantilever oscillations variance signal to assess the viability of the attached organisms. In this contribution, we demonstrate that a more precise analysis of the motion pattern of the cantilever can unveil relevant medical information about bacterial phenotype. We used *B. pertussis* as the model organism, it is a slowly growing Gram-negative bacteria which is the agent of whooping cough. It was previously demonstrated that *B. pertussis* can expresses different phenotypes as a function of the physical-chemical properties of the environment. In this contribution, we highlight that *B. pertussis* generates a cantilever movement pattern that depends on its phenotype. More precisely, we noticed that nanometric scale oscillations of *B. pertussis* can be correlated with the virulence state of the bacteria. The results indicate a correlation between metabolic/virulent bacterial states and bacterial nanomotion pattern and paves the way to novel rapid and label-free pathogenic microorganism detection assays.

## 1. Introduction

In recent years, an increasing prevalence of antimicrobial-resistant pathogens has been reported worldwide [1,2,3,4]. One option to limit their spread is the development of fast and accurate methods and apparatus that could provide, very fast, ideally in real time, antimicrobial sensitivity charts. Few years ago, we demonstrated that Atomic Force Microscopy (AFM) sensors can be used to characterize bacterial susceptibility to antibiotics in a time frame of minutes instead of days as it is the case with traditional techniques [5,6]. The measurement is based on the detection of the nanometric scale oscillations that characterize all living organisms. In practice, the measurement consists of attaching the bacteria of interest onto an AFM cantilever and immersing it in different chemical solutions such as nutrient medium and antibiotics [5,7]. We noticed that the nanometric-scale oscillations of microorganisms are setting the cantilever to oscillate and that these oscillations immediately stop once the attached organisms die. Monitoring the cantilever oscillations as a function of the medium composition present in the analysis chamber leads to antibiotic sensitivity charts in a timeframe of minutes. This nanomotion sensor technique was successfully applied to monitor metabolic activity of microorganisms with a fast and slow growth rate, motile, non-motile, Gram-positive and Gram-negative bacteria in different environmental conditions [7,8,9]. To detect life–death transition or drastic metabolic changes, we performed a simple analysis of the variance of the cantilever oscillations as a function of time. However, this analysis technique did not show any information about more subtle changes occurring in the organism associated with metabolism or gene expression. To expand the application field of nanomotion detection, we implemented a different analysis method that seems more promising. 

The Gram-negative bacterium *Bordetella pertussis* is the etiological agent of whooping cough [10], a highly contagious respiratory tract infection. This pathogen is a slowly growing, Gram-negative aerobic bacterium of 400 nm in width and an average length of 800 nm. The expression of the majority of virulence factors is controlled by a two-component regulatory system (BvgAS) [11]. Depending on the environmental physicochemical properties and the state of BvgAS, *B. pertussis* can adopt different virulence phenotypes. The virulent phase, acquired when BvgAS is active, is defined by the expression of virulence factor genes (adhesins, toxins) and it contributes to pathogenesis. The avirulent phase, when BvgAS is inactivated, could be associated with the need to evade antibodies, facilitate transmission and with the protection against environmental changes and adaptations to ensure survival outside the human host [12,13]. Currently, the methods to evaluate the *B. pertussis* virulence state (i.e., phenotypic analysis by growing bacteria in solid media, real-time polymerase chain reaction, microarray or RNA sequencing techniques) are complex and time consuming [14,15]. Since the metabolic activity and gene set expression of *B. pertussis* cells are documented to be different in the virulent and the avirulent phases [12,13,14,15], we decided to explore the fitness of nanomotion patterns to discriminate in real time both virulence phenotypes. 

## 2. Materials and Methods

### 2.1. Bacteria and Culture Conditions

*B. pertussis* Tohama I reference strain and *B. pertussis* 537 strain, an avirulent phase locked mutant [16], were used. Stock cultures of both strains were stored at −80 °C in 40% (*v*/*v*) glycerol-supplemented culture medium. In preparation for the experiments, these bacteria were grown on Bordetella agar plates with charcoal supplemented with 7% horse blood (BD Difco, Le Pont de Claix, France), for 72 h at 37 °C. Colonies were cultured for another 48 h and then inoculated into 50 mL Erlenmeyer flasks containing 30 mL of Stainer-Sholte (SS) [17] liquid medium and incubated for 24 h at 37 °C on a rotatory shaker (160 rpm).

Last, bacteria were washed three times in phosphate-buffered saline (PBS, pH 7.4) (Sigma-Aldrich, St. Louis, MO, USA), between each rinse they were sedimented by centrifugation at 8500 rpm for 5 min and the pellets were finally suspended in SS liquid medium to obtain a final concentration of 10^6^–10^8^ CFU. For comparative studies where the virulent phenotype is modulated to the avirulent phase, *B. pertussis* Tohama I strain was incubated in SS liquid medium supplemented with MgSO_4_ (Sigma-Aldrich, St. Louis, USA) to a 50 mM final concentration (modulator agent of virulence state). 

### 2.2. AFM Cantilever Preparation

For these experiments, commercially available AFM cantilevers (SD-qp-CONT tipless cantilevers, NanoandMore, GmbH, Wetzlar, Germany) and nanomotion detectors developed in our laboratory were used to perform the nanomotion detection. The cantilevers had a rectangular shape, a length of 130 μm, a nominal resonance frequency of 32 kHz, and a force constant of 0.1 N/m. They had a partially coated end with 60 nm Au, to minimize bimetal bending that might occur upon temperature changes. The cantilever holding chip was deposited onto a dedicated silicon support chip (SD-Align, Nanosensors, NanoWorld AG, Neuchâtel, Switzerland) to simplify the laser adjustment procedure after cantilever replacement. The sensors were incubated for 5 min with a drop (20 µL) of poly-L-lysine solution 0.1% (*w*/*v*) (Sigma-Aldrich, Darmstadt, Germany). Then, they were allowed to air dry for 3 min and incubated with the bacterial suspension for 40 min at 37 °C. Three washes with PBS were performed to eliminate the non-adhering or poorly adhered bacteria. Finally, the cantilever loaded with bacteria was placed on the silicon support chip and immediately introduced into the analysis chamber of the nanomotion detector. The analysis chamber was finally filled with the liquid culture medium (SS liquid medium) to carry out the measurements.

### 2.3. Measurements

Before starting the measurements, the analysis chamber was allowed to stabilize for a short time, (typically less than 5 min were needed) in the liquid medium that was previously filtered using a 0.22 µm pore size filter and brought at room temperature in order to avoid small particles interferences and minimize the thermal drift of the cantilever. The measurements were performed using a custom homemade device developed in the Laboratories of Living Matter Physics at Ecole Polytechnique Federale de Lausanne (EPFL, Lausanne, Switzerland). The device consists of one analysis chamber having a capacity of 2 mL, a super-luminescent light emitting photo-diode to illuminate the cantilever, a four-segment photo-detector and a dedicated electronics equipment that sends the cantilever oscillation signal to a computer using a USB-4431 DAQ card (National Instruments, Austin, TX, USA). For every experiment, two such detectors were used simultaneously to perform duplicated experiments or to compare virulent to avirulent strains on-line and to ensure similar environmental conditions for both samples. 

Homemade software written in LabVIEW (National Instruments, Austin, TX, USA) [18] was used to adjust data acquisition parameters. Typically, the deflection signal was collected at a sampling frequency of 20 kHz for a standard recording duration of 30 min. Each experiment was repeated at least three times. 

### 2.4. Microscopic Characterization of the Bacteria on the Cantilever 

**Figure 1:**
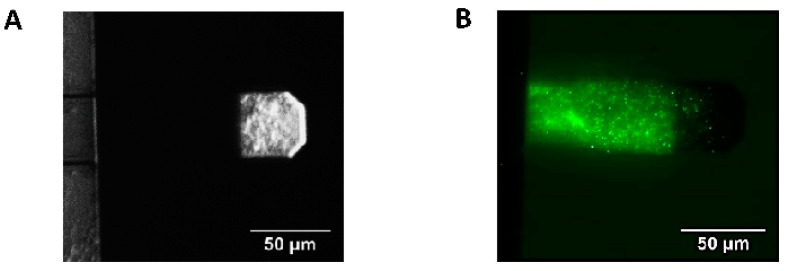
AFM cantilever with *B. pertussis* cells attached. (**A**) Optical image of attached *B. pertussis* 537 strain (avirulent) to the AFM cantilever, the cells are observed on the cantilever section covered by gold. (**B**) Fluorescence microscopy image of an AFM cantilever with *B. pertussis* Tohama I strain (virulent) attached, SYTO 9 Green Fluorescent Nucleic Acid Stain. In this case, only bacteria attached to the non-gold-coated segment of the cantilever are visible.

The device used to perform the experiments was equipped with an optical microscope and a top-mounted camera to monitor microorganisms attached onto the cantilever during the experiments. After each experiment, the cantilever was extracted from the measuring device and a SYTO-9 Green Fluorescent Nucleic Acid Stain (Thermo Fisher Scientific, Waltham, MA, USA) was performed to assess by florescent microscopy that *B. pertussis* cells were still firmly attached to the cantilever. Figure 1 shows typical images of the bacteria-loaded cantilever.

### 2.5. Data Processing

Nanomotion measurements of 30 min recordings were separated in files of 5 min and each of these chunks were analyzed separately. The data was processed with a homemade dedicated MATLAB v2013 program and it was plotted in a chart of 10-s long consecutive violins plots for 5 min. The data processing consisted in dividing the signal, i.e., the z displacement of the cantilever, in 10-s long chunks and display these signal chunks as violin plots. From each of them, we subtracted a linear fit of the signal to get rid of the large thermal movements of the cantilever that we do not consider as biologically relevant. Eventually, the vertical displacements of the cantilever were displayed in histograms. Each column of the histogram represents how many times the cantilever moved a given distance during the duration of the chunk. Finally, to increase readability, the analysis results were displayed as violin plots. The violins plots were displayed by incorporating in our home-made software a freely available MATLAB function: violin.m [19]. Statistical significance was determined using the Wilcoxon test. Error bars represent the standard error (SEM). 

## 3. Results

**Figure 2:**
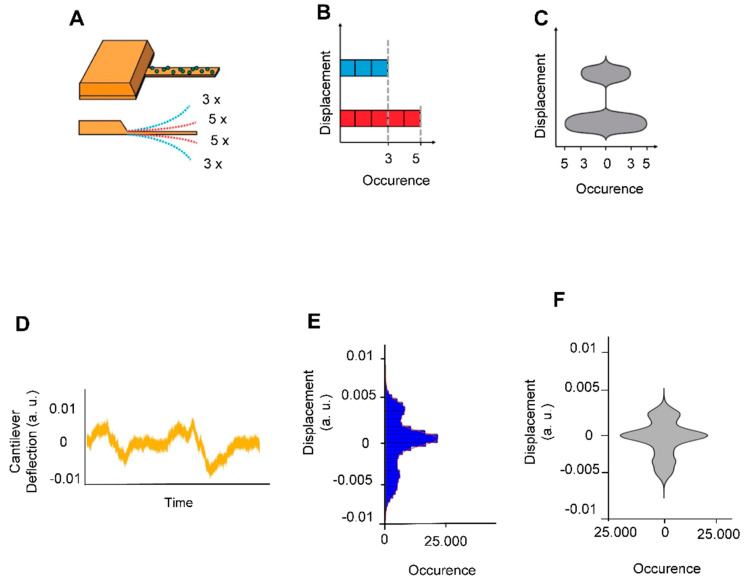
Explanation of cellular nanomotion spectrograms, vertical displacements of the cantilever were displayed as violin plots. (**A**) The cantilever with *B. pertussis* attached (upper panel). In the depicted example, the cantilever deflects 5 times to a short distance (red dashed lines) and 3 times to a larger distance (blue shed lines) during a given time-lapse (lower panel). (**B**) The number of displacement occurrences is plotted as a histogram. (**C**) The histogram is redrawn as a vertical violin plot (referred to as nanomotion spectrogram) in which the amplitude of the cantilever motion is displayed along the vertical axis whereas the number of motion occurrences is displayed symmetrically along the horizontal axis of the graph. The lower panels display (**A**) a typical signal of *B. pertussis* loaded cantilever (**D**) and its corresponding (**E**) histogram and (**F**) spectrograms.

The experiments consisted in attaching living *B. pertussis* cells onto AFM cantilevers and in monitoring the cantilever oscillations as a function of time in liquid culture medium. Figure 2 depicts how such plots were calculated. These plots are referred to as nanomotion spectrograms, and they represent how many times (x axis of the spectrogram) the cantilever moved a given distance (y axis of the spectrogram). In other words, they show the displacement speed distribution in a given period of time. The 10-s period was chosen after several trial-error attempts to find the strongest “contrast” in the spectrograms between virulent and avirulent phases. 

**Figure 3:**
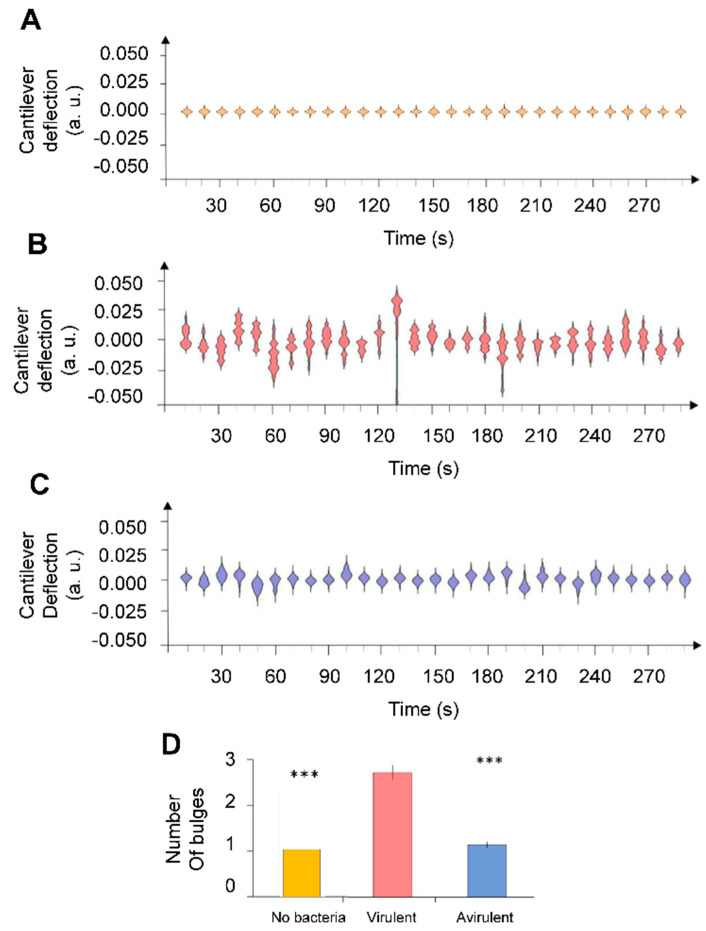
Nanomotion spectrograms of (**A**) control experiment (without bacteria), (**B**) virulent and (**C**) avirulent *B. pertussis* Tohama I strain. Every single spectrogram displays the number of displac-ment occurrences of the cantilever during a time-lapse of 10 s. (**D**) Average number of bulges counted each 10 s during 5 min. Error bars represent standard error whereas stars reflect Wilcoxon test results (*** *p* < 0.01).

Then, we analyzed the metabolic activity of *B. pertussis* cells in both virulent and avirulent phases. Figure 3 represent some typical nanomotion spectrograms recorded without bacteria and with *B. pertussis* in two different virulent phases. Interestingly, nanomotion spectrograms displayed striking differences between virulence states of *B. pertussis* cells. The shape of the avirulent cells spectrograms looks much simpler than those of the virulent variant. The latter possess several bulges and are referred to as multi-modals, contrary to the first ones that in majority only possess one bulge and are mono-modals. Importantly, the presence of multi-modal spectrograms indicates that the cantilever motions are not random but some well-defined displacements occur more frequently than others do. In order to classify the nanomotion spectrograms according to their complexity, we counted the number of bulges. This number is significantly higher in spectrograms recorded with virulent *B. pertussis* cells than with their avirulent counterparts (Figure 3D), suggesting a different vibration pattern of the virulent specimens.

**Figure 4:**
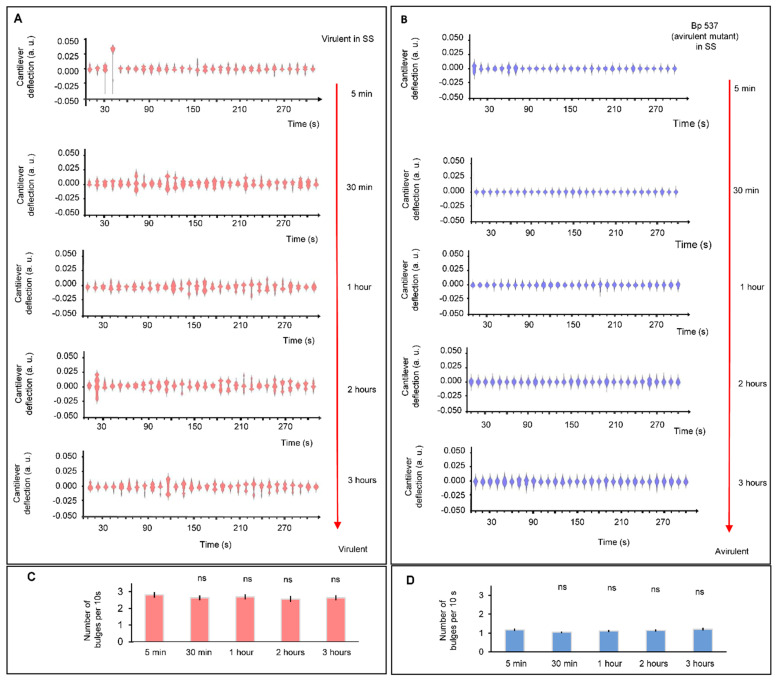
Comparative image of nanomotion spectroscopies. (**A**) Virulent *B. pertussis* Tohama I incubated in conditions that maintain the virulent phase at 5 and 30 min, 1, 2 and 3 h. (**B**) Avirulent *B. pertussis* 537 strain at 5 and 30 min, 1, 2 and 3 h. Every single spectrogram displays the number of displacement occurrences of the cantilever during a time-lapse of 10 s. Bar chart shows the mean number of bulges for 10 s in each period of time to (**C**) virulent *B. pertussis* Tohama I and (**D**) avirulent *B. pertussis* 537. Error bars represent standard error for 30 different periods of 10 s. The Wilcoxon test did not highlight significant differences (ns: not significant).

To evaluate the stability of the virulence states in the absence of external stimuli we conducted long measurements in steady state conditions with both virulent and avirulent phenotypes. A nanomovement evaluation study was carried out over 3 h with the virulent *B. pertussis* Tohama I (Figure 4A,C) and the avirulent mutant strain *B. pertussis* 537 (Figure 4B,D), which confirmed that the virulent and avirulent phase bacteria exhibited a constant vibration pattern during the experiment. 

**Figure 5:**
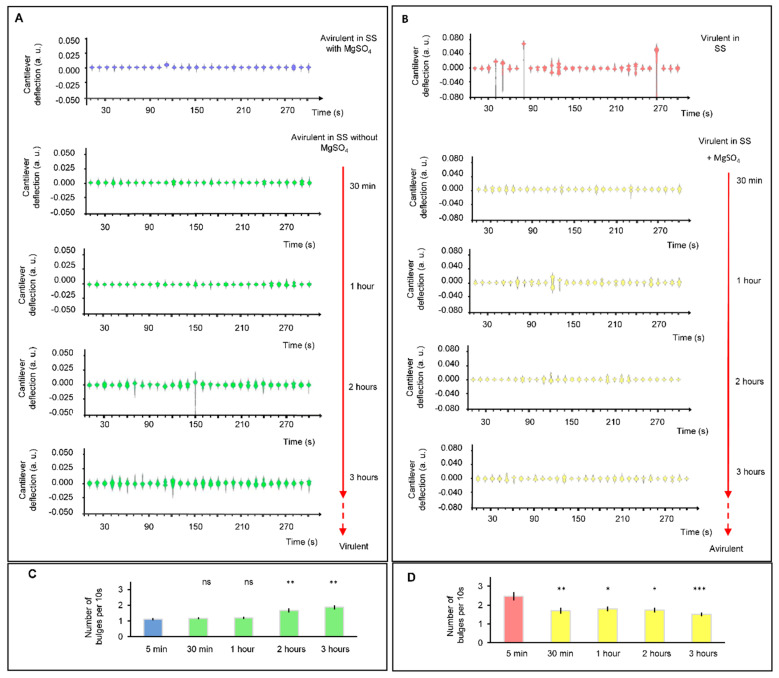
Cellular nanomotion spectrograms of *B. pertussis* cells in the transition between virulence phases. (**A**) Nanomotion of avirulent cells under conditions that induce a virulent phase (without MgSO_4_ in the liquid medium); at 30 min, one, two and three hours. (**B**) Nanomotion of virulent cells under conditions that induce an avirulent state (SS liquid medium with 50 mM MgSO_4_ in chamber) at 30 min, one, two and three hours. Every single spectrogram displays the number of displacement occurrences of the cantilever during a time-lapse of 10 s. (**C**) Bar chart shows the mean number of bulges of 10 s violins during the transition from avirulent to virulent. (**D**) Bar chart shows the mean number of bulges of 10 s violins during the transition from virulent to avirulent phases. Error bars represent standard error for 30 different periods of 10 s. The Wilcoxon test as compared to the initial condition (5 min): *** *p* < 0.001; ** *p* < 0.01; * *p* < 0.05; ns: not significant.

Finally, we carried out experiments that consisted of inducing a modulation or transition of *B. pertussis* cells from avirulent to virulent phase (Figure 5A) and vice versa (Figure 5B). For this purpose, we used MgSO_4_ (50 mM), which switches the BvgAS from active to inactive state. The observed difference between the nanomotiom spectrogram shapes of virulent and avirulent B. pertussis are probably representing dissimilar metabolic activities and/or gene expression. It can be noticed that significative changes in the spectrogram shapes (and number of bulges) in the avirulent to virulent phase (Figure 5C) were registered after 2 h, but the nanomotion changes during virulent to avirulent phase transition (Figure 5D) occurred more rapidly. Our results show that after 30 min of incubation, there is a significant reduction in the cantilever nanomotion of the bacteria in the presence of the modulating agent. These results agree with those reported by Metz et al. [20] who evaluated the response of *B. pertussis* virulent phase in the presence of 50 mM MgSO_4_ via proteomic analysis. Therefore, shutting down metabolic processes associated with the expression of virulence factors is quicker than starting new ones from the avirulent state. During the transition from the virulent to avirulent phase, *B. pertussis* simultaneously loses the ability to synthesize several virulence-associated factors such as adhesins, toxins and other factors that allow bacteria to achieve a successful infection. These factors include pertussis toxin (Ptx), fimbriae (Fim), adenylate cyclase toxin (ACT), hemolysin (Hly), dermonecrotic toxin (DNT), pertactin (Prn), filamentous hemagglutinin (FHA), and cytochrome d-629 [21]. Additionally, it has been mentioned that genes of secretion system proteins and membrane-associated transporter proteins are less expressed in the avirulent state [13]. Rapid reprogramming of metabolism and gene expression towards the acquisition of the avirulent phase could help to have a fast response to environmental and nutritional stress and could be an advantage for bacterial persistence in the host [13,22,23]. 

For the modulation from the avirulent to the virulent phase, temporal regulation of the virulence genes has been reported [24]. Virulence activated genes (vags) have been categorized into three temporal classes: early, intermediate and late genes. Early genes include those that are activated rapidly in response to non-modulation conditions; intermediate genes are expressed about 1 h after the activation of the BvgAS locus; and late genes are activated 2–4 h after the activation of the BvgAS locus. In our experiments, the results of the time-course of cantilever movements are detected after 2 h of incubation of avirulent bacteria under non-modulating conditions. This result is consistent with that reported by Veal-Carr and Stibitz [24] on expression kinetics of virulence factors of *B. pertussis* where full expression starts around 2 h after exposition of avirulent bacteria to non-modulation conditions. Therefore, significant differences could reflect the temporal-full expression of vags genes mentioned before. It is interesting to point out that during the modulation from the avirulent to the virulent phase, the rise in the number of bulges, from 1.1 (±0.07) to 1.87 (±0.14) after 3 h of incubation, would reflect the increase in the virulence factors synthesis until it reaches the maximum expression at a later step.

Importantly, frequency domain analysis (FFT) of the signal recorded with virulent and avirulent samples were very similar (data not shown) and did not show any characteristic frequency that could be specifically attributed to virulent or avirulent phenotypes.

## 4. Discussion

The results presented here indicate that nanomotion spectroscopy technique can be very useful to distinguish rapidly between different physiological states such as the virulence of bacteria. This analysis technique highlighted differences in the nanosensor motion pattern between *B. pertussis* in its virulent and avirulent phases. The complex shape of nanomotion spectrograms reflects the different cellular activity of the both phenotypes. Gene expression and metabolic pathways are different in avirulent and virulent B. pertussis. Additionally, different proteins are expressed on the surface of the two phenotypes [11,13]. The origin of the nanomotion is unfortunately still partially unknown, however several evidences point out that different metabolic levels [5] and/or protein conformation changes [25] induce detectable cantilever oscillations. We, therefore, suspect different metabolic states and/or different membrane protein conformation changes to be at the origin of the differences in nanomotion pattern of the two phenotypes. Correlating nanomotion patterns to specific metabolic/physiological states of bacterial cells opens the way to a better understanding of the different metabolic activities of biological systems. An additional advantage of such this type of sensors is the limited number of microorganisms needed to generate a signal (50–500 bacteria, less than 5 yeast cells and one single mammalian cell). In order to extend the application of this technique, we are planning a future experiment that will involve pathogens with different invasive potential. Correlating pathogenic potential to nanomotion spectrograms could lead to a new generation of ultra-rapid, label-free diagnostic devices. Combining this nanomotion technique with conventional metabolic assays will lead to investigation procedures of metabolic changes in living systems with an unprecedented temporal resolution. 

## Data Availability

The data presented in this study are available on request from the corresponding author.
